# Non-COVID-19 Vaccinations and the Induction of Autoantibodies in Pemphigus Diseases: A Review of the Speculative Issue and Our Clinical-Laboratory Experience

**DOI:** 10.3390/antib15020033

**Published:** 2026-04-10

**Authors:** Maksymilian Markwitz, Natalia Welc, Klementyna Kępińska, Monika Bowszyc-Dmochowska, Marian Dmochowski

**Affiliations:** 1 Autoimmune Blistering Dermatoses Section, Department of Dermatology, Poznan University of Medical Sciences, 60-355 Poznan, Poland; 2 Doctoral School, Poznan University of Medical Sciences, 60-812 Poznan, Poland; 3 Cutaneous Histopathology and Immunopathology Section, Department of Dermatology, Poznan University of Medical Sciences, 60-355 Poznan, Poland

**Keywords:** pemphigus diseases, vaccination, autoantibodies

## Abstract

**Background**: Pemphigus diseases are rare autoimmune blistering disorders mediated by pathogenic autoantibodies directed mainly against desmoglein 1 and desmoglein 3. Although most cases are considered idiopathic, external triggers that can disrupt immune tolerance have been described. Vaccination has been discussed as a potential precipitating factor in autoimmune skin diseases. However, the relationship between vaccination and the induction of pemphigus-related autoantibodies has not been comprehensively summarized. **Methods**: We conducted a narrative review of all available studies published in the last 25 years identified through medical databases, excluding studies on COVID-19 vaccinations. Reports describing either new-onset pemphigus or exacerbation of preexisting pemphigus with a temporal association to vaccination were included. Clinical characteristics, vaccine type, latency period, direct immunofluorescence findings, and ELISA results for desmoglein autoantibodies were analyzed. In addition, we present our own clinical-laboratory experience illustrating this issue. **Results**: The current evidence consists predominantly of case reports and small case series. Published cases describe pemphigus vulgaris and pemphigus foliaceus occurring after vaccinations against influenza, hepatitis B, tetanus, diphtheria, pertussis, rabies, and other routinely administered immunizations. The latency period most often ranged from several days to a few weeks. Immunopathological findings were consistent with classical pemphigus diseases, including intercellular IgG deposits in the epidermis and circulating autoantibodies against desmoglein 1 and/or desmoglein 3. Our patient was a 78-year-old woman who developed cutaneous form of pemphigus vulgaris, diagnosed with direct immunofluorescence (DIF) and multiplex ELISA, 10 days after diphtheria–tetanus–pertussis vaccination. The patient had a positive family history of autoimmune blistering disease, namely mucous membrane pemphigoid. **Conclusions**: Based on the currently available evidence, a direct causal relationship between vaccination and pemphigus diseases cannot be established. Nevertheless, accumulated clinical and serological observations suggest that vaccination may act as a triggering factor in genetically or immunologically predisposed individuals, possibly by amplifying pre-existing subclinical autoreactive immune responses. Further population-based and mechanistic studies are required to clarify this association, while the overall benefits of vaccination remain substantial.

## 1. Introduction

The term “autoimmune pemphigus diseases” (APDs) refers to a group of autoimmune blistering disorders that include pemphigus vulgaris (PV) and pemphigus foliaceus (PF) [1,2]. These entities are characterized by autoantibody-mediated damage directed against structural epidermal proteins. In mucosal PV, autoantibodies primarily target desmoglein-3 (DSG3), whereas in PF, they target desmoglein-1 (DSG1). In mucocutaneous PV, both DSG1 and DSG3 are involved [1,2,3]. Pemphigus diseases are prototypical antibody-mediated disorders in which pathogenic IgG autoantibodies, predominantly of the IgG4 subclass, bind to desmosomal cadherins responsible for keratinocyte adhesion [2,4]. Binding of anti-DSG1 and anti-DSG3 antibodies disrupts desmosomal integrity. This process involves both direct impairment of intercellular adhesion and activation of intracellular signaling pathways, including p38 MAPK, Src, and EGFR, ultimately leading to desmosomal disassembly and acantholysis [5,6]. Increasing evidence indicates that pemphigus is not solely an antibody-driven disorder. Its pathogenesis also involves complex interactions between T and B lymphocytes, with a predominance of Th2-type immune responses characterized by elevated levels of cytokines such as IL-4, IL-10, and IL-21 [4,7]. Despite extensive research, the precise mechanisms responsible for the initial loss of immune tolerance remain incompletely understood [2,4].

Pemphigus diseases are considered rare illnesses, with incidence varying across geographic regions and ethnic populations [8,9]. Despite their low frequency, they carry substantial clinical significance. Pemphigus diseases are characterized by astonishingly diverse clinical presentations [10]. Before the introduction of systemic corticosteroids, pemphigus diseases were frequently fatal [11]. Although modern immunosuppressive and biologic therapies have markedly improved patient outcomes, pemphigus diseases remain associated with considerable morbidity and long-term treatment burden, including the risk of serious adverse events [8]. Early diagnosis supported by appropriate laboratory techniques, including indirect immunofluorescence (IIF) and enzyme-linked immunosorbent assays for anti-desmoglein antibodies, is therefore essential [8,12]. While most cases of pemphigus diseases are regarded as idiopathic, increasing attention has been directed toward induced forms of them, including drug-induced autoimmune pemphigus (DIAPD) [13]. Drug-induced autoimmune pemphigus (DIAPD) is a well-recognized variant of pemphigus in which systemic medications can initiate or exacerbate disease. Several pharmacological agents, particularly those containing thiol (sulfhydryl) groups such as penicillamine, captopril, and bucillamine, have been consistently reported as triggers, although many other drugs across different chemical classes have also been implicated [13,14]. The clinical and immunopathological features of DIAPD are often indistinguishable from idiopathic pemphigus, with circulating and tissue-bound IgG autoantibodies against desmogleins, making differentiation based on phenotype alone challenging [13,14]. Identification and withdrawal of the offending drug may lead to disease remission in a substantial proportion of cases, supporting the role of pharmacological triggers in susceptible individuals [13]. These observations reinforce the concept that external immune stimulation can, under certain circumstances, contribute to the breakdown of self-tolerance and autoimmune blistering.

Pemphigus diseases have also been reported following vaccination and may represent a potential trigger or precipitating factor in susceptible individuals [9,13]. Notably, observations suggesting a possible association between vaccination and pemphigus diseases are not new. One of the earliest reports, published in 1963 before the dawn of immunofluorescence, described an unusual case of cutaneous spreading of pemphigus vegetans following immunization [15]. Although considered rare, such observations have gained attention in the context of widespread immunization programs and the introduction of novel vaccine platforms [16]. Vaccines are designed to induce controlled activation of innate and adaptive immune responses through antigen presentation, co-stimulatory signaling, and cytokine production [17]. mRNA- and viral vector-based vaccines are associated with strong type I interferon responses and enhanced immune activation [17,18]. In genetically or immunologically predisposed individuals, this stimulation may theoretically contribute to a breakdown of self-tolerance through mechanisms such as molecular mimicry, epitope spreading, bystander activation, or polyclonal B-cell stimulation [19,20]. However, current evidence is largely based on case reports and small case series, and it remains unclear whether vaccination represents a true etiological trigger, an accelerator of subclinical autoimmunity, or merely a temporal association [9,13].

Given the increasing number of reports describing a temporal relationship between vaccination and the onset or exacerbation of pemphigus diseases, careful clinical and laboratory evaluation of such cases is warranted. As COVID-19 vaccination has already been extensively reviewed in previous publications, we focused on cases associated with other routinely administered vaccines [21]. To our knowledge, no previous review has specifically addressed this topic. The aim of the present study was to retrospectively analyze patients in whom a vaccine-related association was suspected, assess changes in autoantibody levels, and discuss potential immunopathogenic mechanisms in the context of currently available evidence.

## 2. Methodology

**Figure 1:**
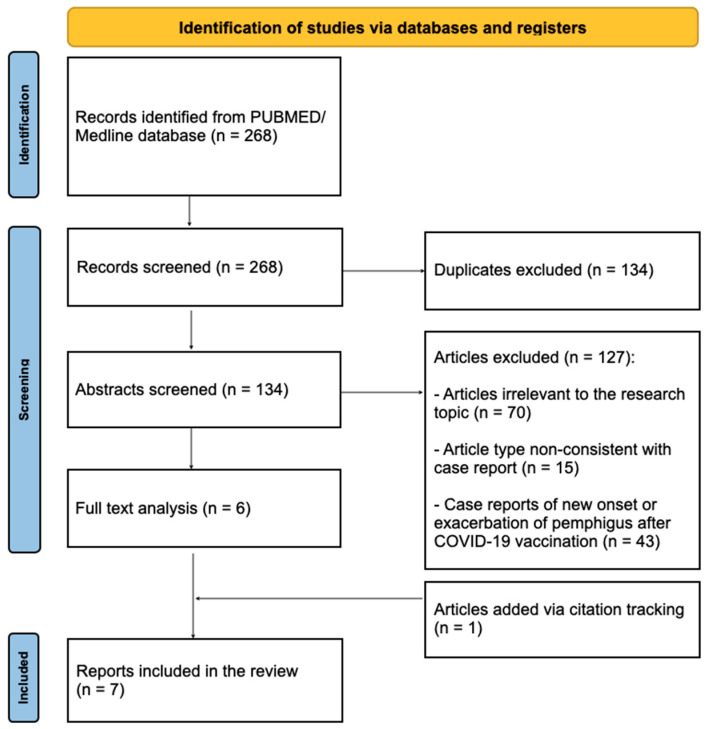
The screening and selection process, according to the PRISMA guideline.

The authors conducted a literature review of articles available in the PubMed/MEDLINE database from the last 25 years. The search keywords were “pemphigus, vaccine” and “pemphigus, vaccination”. In total, 268 papers were identified. Only case reports reporting new onset or exacerbation of pemphigus after vaccination other than against COVID-19 were included in the review. After excluding duplicate reports of pemphigus after COVID-19 vaccinations, or articles of types other than case reports or case series, six remaining papers were analyzed in terms of potentially causative vaccines, detailed patient information, and specific data concerning the clinical picture, diagnosis, and treatment. One article not indexed in PubMed was added manually via citation search. Finally, seven articles were selected for review (Figure 1).

## 3. Results

### 3.1. Reported Cases in the Literature

Analysis of seven identified cases of pemphigus diseases temporally associated with vaccination, including the patients’ sex and age, diagnosis, vaccine, clinical picture, and treatment, is presented in Table 1.

### 3.2. Our Clinical-Laboratory Experience

**Figure 2:**
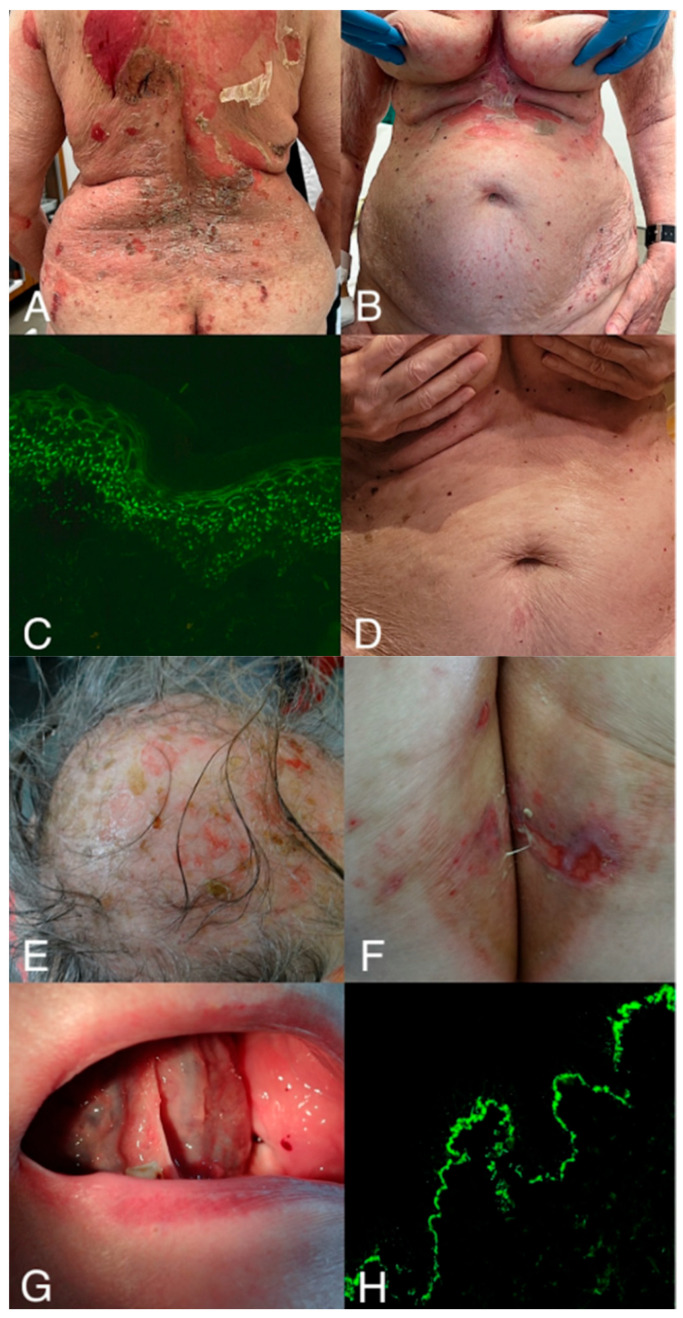
An elderly female with cutaneous form of pemphigus vulgaris following a recent vaccination against diphtheria, tetanus, and pertussis (DTP vaccine). (**A**,**B**)—A 78-year-old woman was admitted to the Dermatology Clinic in University Hospital in Poznań, Poland, due to a massive bullous skin eruption accompanied by severe itching, which started 2 weeks prior to admission. The patient’s comorbidities were hypertension and degenerative joint disease. Her only permanent drug was metoprolol. Additionally, the patient was vaccinated with the DTP vaccine 10 days before initial cutaneous lesions had developed. Upon admission, a dermatological examination revealed numerous large erosions on the chest, back, and upper limbs; Nikolsky’s sign was positive. In gynecological and otorhinolaryngological consultation, mucosal lesions were excluded. (**C**)—Direct immunofluorescence (blue light-emitting diode technology-operated microscopy, Euroimmun, Lübeck, Germany) showed pemphigus IgG4 deposits (+++) having dew drops on a spider web appearance in the epidermis. Moreover, the pemphigus IgG1 (+++) and C3 (+) deposits were also found in the epidermis, more prominent in its lower part. The six-parameter ELISA panel was positive towards desmoglein 1, desmoglein 3, and BP180. Additionally, given the patient’s age and clinical presentation, a paraneoplastic workup was performed, which yielded no findings suggestive of an underlying malignancy. Diagnosis of the cutaneous form of pemphigus vulgaris was established. (**D**) Methylprednisolone was initially administered intravenously and then orally in tapering doses; ceftriaxone was administered intravenously; clobetasol propionate and fusidic acid were administered topically, resulting in improvement of the skin lesions. The patient was later qualified for the rituximab therapy. Remission of skin lesions was observed 3 months after rituximab treatment. The link between DTP vaccination and the development of pemphigus vulgaris remains uncertain. (**E**–**G**)—Moreover, the patient’s family history of autoimmune blistering diseases was positive—the patient’s sister has mucous membrane pemphigoid, diagnosed also at the age of 78. (**H**) In her case, direct immunofluorescence (laser-scanning confocal microscopy, Carl Zeiss Jena GmbH, Jena, Germany; photomicrograph courtesy of Professor Justyna Gornowicz-Porowska, MSc, PhD) showed IgG4 (+++) deposits in an indeterminate pattern along the dermal-epidermal junction. Monoparametric ELISA was positive towards BP180. Both photomicrographs were taken at the original objective magnification ×40.

The clinical presentation of the patient is shown in Figure 2.

## 4. Discussion

As presented in Table 1, several case reports published prior to the COVID-19 pandemic have described the occurrence or exacerbation of pemphigus following vaccinations against various infectious diseases. These observations suggest that vaccination may serve as an environmental trigger in susceptible individuals, although a causal relationship remains difficult to establish.

De Simone et al. reported an exacerbation of pemphigus after influenza vaccination, highlighting that environmental factors such as infections, drugs, trauma, or immunizations may contribute to disease onset or flares in genetically predisposed patients [27]. Other reports describe the development of pemphigus shortly after administration of different vaccines. A case of pemphigus following tetanus–diphtheria immunization occurred in an 11-year-old girl who developed cutaneous lesions approximately one week after vaccination. Histopathology revealed suprabasal acantholysis, and direct immunofluorescence demonstrated intercellular IgG and C3 deposition in the epidermis, findings consistent with pemphigus. The authors suggested that vaccine components containing thiol groups might have triggered the autoimmune process in this patient [23]. Additional reports have documented similar temporal associations with other vaccines, including influenza, anthrax, and hepatitis-related immunizations, as well as pre-travel vaccinations [24,25,26,27,28]. In these cases, patients developed either new-onset pemphigus or exacerbations of pre-existing disease within days to weeks after vaccination.

The temporal relationship suggested that immune activation induced by vaccination might have served as a precipitating factor. Possible contributions to this immune activation include vaccine adjuvants. Autoimmune Syndrome Induced by Adjuvants (ASIA) develops after exposure to adjuvants such as vaccinations, silicone, mineral oils, and paraffin, as well as other substances with adjuvant properties [16]. Adjuvants are used in vaccines to enhance the immune response. In theory, they may contribute to the breakdown of immune tolerance in predisposed individuals. They activate the innate immune response and induce cytokine production, which may promote mechanisms such as molecular mimicry, bystander activation, and polyclonal B-cell activation. It should be emphasized that the available data are primarily indirect and do not permit the establishment of a causal relationship. The role of adjuvants in the pathogenesis of pemphigus diseases remains hypothetical and requires further investigation [16]. Moreover, in a cohort of Danish and Swedish adult women vaccinated against Human Papilloma Virus (HPV), pemphigus vulgaris was identified as one of the diseases with a statistically significantly higher risk of occurring within 179 days of HPV vaccination (RR = 8.75, 1.04–73.99) [29].

Interestingly, while the Bacillus Calmette–Guérin (BCG) vaccine has not been reported as a potential trigger for pemphigus in the literature, a case report describes a patient with non-muscle-invasive bladder cancer who developed pemphigus vegetans following local BCG immunotherapy. The authors suggest that intravesical BCG therapy may induce pemphigus vegetans by promoting immune activation via cytokine release and T-cell stimulation. This activation could lead to the production of autoreactive CD4^+^ lymphocytes that target desmogleins, resulting in the formation of pathogenic anti-desmoglein (DSG) autoantibodies [30].

In the present study, we analyzed the possible association between vaccinations other than COVID-19 and the induction of pemphigus. Reports of autoimmune blistering diseases following vaccination are not new and were described long before the pandemic. These cases involved, among others, vaccines for influenza, hepatitis B, tetanus, and rabies [22,23,24,25,26,27,28,29,30]. It should be emphasized, however, that such cases are rare and most available data derive from isolated case reports or small case series [31]. To date, no population-based studies have clearly demonstrated an increased incidence of pemphigus following vaccination. In one study, the incidence of influenza was higher in the vaccinated group than in the unvaccinated group, but this difference did not reach statistical significance [32].

Analogous to drug-induced pemphigus, vaccination may act as an immunological stimulus that reveals disease in susceptible individuals [31]. In DIAPD, the external factor does not create a new disease entity. Instead, it may disrupt immune balance and promote loss of tolerance to antigens characteristic of pemphigus, mainly desmoglein 1 and desmoglein 3 [33]. In most cases, the autoantibody profile is similar to that observed in idiopathic forms. However, the spectrum of target antigens may be broader, including other structural proteins involved in cell adhesion. In our patient, in addition to anti-desmoglein antibodies, anti-BP180 antibodies were also detected. The presence of anti- BP180 antibodies should be considered in the differential diagnosis, particularly in the context of possible overlap syndromes between pemphigus and pemphigoid. In the present case, the clinical presentation and immunopathological findings, including no immune deposits along the dermal–epidermal junction (DEJ) in direct immunofluorescence, were most consistent with pemphigus. However, the detection of anti-BP180 antibodies in an elderly individual with a family history of the positivity of BP180 antibodies may reflect coexisting autoreactivity related to epitope spreading. We believe that, in general, in elderly people, the presence of serum antibodies against BP180 alone without the detection of immune deposits along the DEJ in tissues should not be considered diagnostic for pemphigoid diseases, because it is likely just a feature of senescence of the immune system. Overlapping serological profiles have been described in the literature and do not always correspond to a fully developed clinical phenotype of another autoimmune blistering disease [33]. This may reflect non-specific polyclonal B-cell activation in response to strong immune stimulation. Strong activation of innate and adaptive immune responses may amplify pre-existing, subclinical autoreactivity [34]. In this context, vaccination should be considered as temporally associated with the disease rather than a primary cause.

The mechanisms that may explain the development of autoimmune disorders after vaccination remain under debate. Molecular mimicry is most frequently proposed [34]. This concept assumes structural similarity between vaccine antigens and host proteins. In such a situation, an immune response directed against an infectious antigen could lead to cross-reactivity with self-structures. In pemphigus, this would theoretically involve desmosomal proteins such as desmoglein 1 and desmoglein 3 [33]. However, direct cross-reactivity between classical vaccine components and these antigens has not been demonstrated. Another proposed mechanism is bystander activation. Strong immune stimulation following vaccination leads to cytokine release and activation of antigen-presenting cells [19,34]. In genetically predisposed individuals, this may promote activation of previously silent autoreactive T and B lymphocytes. Subsequently, epitope spreading may occur. This mechanism is well recognized in autoimmune diseases and may also contribute to the pathogenesis of pemphigus [19,34].

Vaccination activates innate immunity through Toll-like receptors and induces type I interferon production [19]. Type I interferon enhances antigen presentation, B-cell differentiation, and antibody production. In pemphigus, Th2, Th17, and T follicular helper responses play an important role [7]. Polyclonal B-cell activation may increase antibody production, including autoantibodies against desmogleins [4]. According to current knowledge, vaccination may act as a trigger, primarily in individuals with underlying genetic or immunological susceptibility, including pre-existing subclinical autoreactivity [31]. Such susceptibility may be related to specific HLA alleles, cytokine gene polymorphisms, or impaired immune regulation [35]. There is no evidence that vaccination alone induces de novo autoimmune responses in individuals without this background.

In our clinical case, a clear temporal association was observed between DTP vaccination and the onset of cutaneous symptoms. The first lesions appeared 10 days after vaccination, which falls within the latency period described in isolated reports of pemphigus following diphtheria or tetanus-containing vaccines. Cases of pemphigus following DTP or related DT vaccines are rare, with only a few reports, including cases following tetanus and diphtheria vaccination and a single report suggesting DTP as a potential trigger of pemphigus vulgaris [23,24,26]. In this context, our observation represents another example of a possible temporal association, although the overall number of documented cases remains very small. Importantly, the patient had a positive family history of autoimmune blistering disease. Her sister was diagnosed with mucous membrane pemphigoid at a similar age. This finding supports the possibility of a genetic susceptibility. Associations between pemphigus and specific HLA class II alleles, as well as polymorphisms in genes such as ST18, FOXP3, and cytokine-related genes, have been described [35,36]. In such a context, vaccination may have acted as a precipitating factor unmasking previously subclinical autoimmunity.

It is also noteworthy that reports of pemphigus following vaccination more frequently describe pemphigus foliaceus than classical mucosal-dominant pemphigus vulgaris [31]. This observation requires systematic evaluation. It would be important to determine whether cases of mucosal-dominant PV with isolated anti-desmoglein 3 antibodies were present. It is possible that post-vaccination immune responses more often involve desmoglein 1 than desmoglein 3, thereby favoring a cutaneous phenotype. The relative contributions of DSG1 and DSG3 to pemphigus pathogenesis depend on their tissue expression patterns. This hypothesis requires confirmation through detailed serological analyses.

The occurrence of pemphigus after vaccination does not by itself prove that the vaccine caused the disease. In most instances, epidemiological data demonstrating an increased incidence of pemphigus after vaccination are lacking. The limited number of reported cases and the absence of controlled studies represent important constraints. The main limitations of our study are the small number of analyzed cases and its observational design. The available literature is predominantly based on single-case reports, with no population-based evidence of an increased incidence. Furthermore, no direct mechanistic proof of causality exists. In the presented case, genetic testing, including HLA typing, was not performed, which precludes assessment of individual immunogenetic susceptibility. For these reasons, the findings should be interpreted with caution.

Temporal associations between vaccination and the occurrence of autoimmune blistering diseases should be interpreted with caution. It should be emphasized that the development of disease after vaccination does not, in itself, constitute evidence of a causal relationship between these events [36]. Autoimmune diseases may occur shortly after vaccination purely by coincidence [37,38]. This may reflect the natural incidence of these disorders in the general population. In addition, increased clinical awareness and reporting bias may contribute to the perception of a stronger association than actually exists. Currently available data suggest that vaccination may occasionally act as a nonspecific immunological stimulus that reveals pre-existing subclinical autoimmunity in predisposed individuals, rather than serving as a primary etiological factor of disease [37,39]. It should also be clearly emphasized that the well-established benefits of vaccination in preventing infectious diseases across entire populations clearly outweigh the potential and still unconfirmed risk of triggering autoimmune blistering diseases [37].

Physicians of various specialties should be aware that pemphigus diseases can be life-threatening. Vaccinations may, in rare instances, be temporally associated with pemphigus diseases in genetically predisposed individuals. However, the currently available evidence is based predominantly on case reports and small case series, and therefore does not establish a direct causal relationship. The benefits of vaccination, particularly in patients receiving immunosuppressive therapy, continue to outweigh the potential risks [31]. Rather than classifying these cases as unequivocally pemphigus temporally associated with vaccination and placing them in a broader category of drug-triggered pemphigus diseases, it may be more appropriate to consider [40] them as rare, potential event associated with vaccination that require further investigation. Vaccinations in the context of triggering pemphigus diseases clearly show the dichotomy between the population and the individual. Further population-based and mechanistic studies are needed to better define the relationship between vaccination and autoimmune blistering diseases. Increased awareness of this possible association may facilitate earlier diagnosis and more timely therapeutic intervention in selected patients. The crucial question of whether it is safe to continue vaccinating individuals who already suffer from pemphigus diseases temporally associated with vaccination remains unresolved. Of note, according to a recent large-scale, population-based cohort study, COVID-19 infection is associated with an elevated risk of autoimmune blistering diseases, whereas COVID-19 vaccination decreases this risk [40].

## 5. Conclusions

In conclusion, based on the currently available data, a direct causal relationship between vaccination and the development of pemphigus diseases cannot be confirmed. The reported cases are sporadic in nature and rely predominantly on clinical observations. Nevertheless, the accumulated evidence suggests that, in rare instances, vaccination may be temporally associated with disease onset in genetically or immunologically predisposed individuals, possibly by amplifying pre-existing subclinical autoreactive immune responses.

There is currently no evidence that vaccination can induce pemphigus diseases in individuals without underlying susceptibility. In most cases, the benefits of vaccination clearly outweigh the potential risks. In selected situations, particularly in individuals with a personal or family history of autoimmune blistering diseases, the decision to vaccinate should be preceded by careful clinical evaluation and an individualized risk–benefit assessment. Further population-based and immunological studies are required to clarify the significance of this association.

## 6. Future Directions

Future studies should aim to clarify the possible relationship between vaccination and the development of pemphigus. Current evidence is mainly based on case reports and small case series. Therefore, large population-based studies are needed to determine whether the incidence of pemphigus is truly increased after vaccination or whether the observed cases represent coincidental temporal associations. Prospective studies with detailed clinical and laboratory follow-up may also be useful. Monitoring of anti-desmoglein antibodies before and after vaccination could help determine whether vaccination induces new autoantibody production or rather amplifies pre-existing subclinical autoreactivity.

Another important direction concerns genetic and familial susceptibility. Our case suggests that individuals with a personal or family history of autoimmune blistering diseases may represent a subgroup with increased vulnerability. Future research should evaluate whether genetic factors, including HLA alleles and other immune-regulatory variants, influence the risk of disease onset following immune stimulation, such as vaccination. At present, it remains unclear whether vaccination increases the risk of pemphigus or whether the temporal associations observed in some patients reflect coincidence. Careful interpretation of such observations is necessary. Importantly, the overall benefits of vaccination remain well established.

## Figures and Tables

**Table 1:** Literature review of available cases of pemphigus diseases temporally associated with vaccination.

Authors (Year)	Patient	Diagnosis	Vaccine	Clinical Picture	Diagnostics	Treatment
Korang et al. (2002) [22]	Female, 45	Exacerbation of pemphigus foliaceus	Tetanus	Erythematous scaly plaques mainly on the trunk, the legs, and the face, three months after the vaccination	Not available	Not available
Cozzani et al. (2002) [23]	Female, 11	New onset of pemphigus (classified as drug-induced)	Tetanus and diphtheria	Vesicular lesions and erosions on the face, trunk, and arms 7 days after vaccination	Histopathology: acantholysis, with suprabasal cleavage. DIF: intercellular deposits of IgG and C3 in the epidermis, granular deposits of IgM at the dermo-epidermal junction and in the vessels of the superficial dermis	No treatment
Berkun et al. (2005) [24]	Male, 43	New onset of pemphigus vulgaris	Hepatitis B Virus (HBV)	Generalized blisters on the face, the trunk, and in the oral mucosa three months after vaccination	Histology, DIF, ELISA—confirmed the diagnosis of pemphigus vulgaris (no specific details)	Prednisone, intravenous immunoglobulins, and azathioprine
Muellenhoff et al. (2004) [25]	Male, 41	New onset of pemphigus vulgaris	Anthrax	Oral mucosa ulcerations on the same day of the vaccination	DIF: strong cell surface IgG deposition, IIF: IgG titers elevated at 1:40, histopathology of the oral cavity: intramucosal blister formation, exocytosis of neutrophils, and acantholysis	Prednisone, azathioprine, cyclosporine
Yalcin et al. (2007) [26]	Female, 43	New onset of pemphigus vulgaris	Rabies	Severe ulcerations in the mouth and ruptured bullous lesions, erosions, and bullae on the body and extremities 10 days after the last shot of the vaccine (0, 7, 21, 28)	DIF: IgG staining detected on the cell surfaces of keratinocytes. IIF with monkey esophagus: revealed IgG staining	Prednisolone, azathioprine, and topical treatment
De Simone et al. (2008) [27]	Male, 47	Exacerbation of pemphigus vulgaris	Influenza	Blisters and erosions disseminated on the trunk, tongue, palate and conjunctiva 7 days after the vaccination	Diagnosis based on previous clinical, histochemical and immunopathological findings	Prednisone, mycophenolate mofetil
Kadylak et al. (2024) [28]	Male, 38	New onset of pemphigus foliaceus	Hepatitis A, rabies, cholera, typhoid fever, and yellow fever	Numerous erosions, crusts, hyper-, hypopigmentation, and single blisters on the trunk and limbs	Histopathology: a slightly loosened structure of surrounding epidermis in the deeper layers without evident acantholysis. The stroma with a fairly intense perivascular lymphocytic infiltrate with occasional eosinophils. DIF: intercellular IgG, C3c, and C1q deposits. ELISA: DSG1 positive	Prednisone, topical treatment

## Data Availability

The authors confirm that the data supporting the findings of this study are available within the article.
